# Shedding light on the ‘dark side’ of phylogenetic comparative methods

**DOI:** 10.1111/2041-210X.12533

**Published:** 2016-06-13

**Authors:** Natalie Cooper, Gavin H. Thomas, Richard G. FitzJohn

**Affiliations:** ^1^ School of Natural SciencesTrinity College DublinDublin 2Ireland; ^2^ Department of Life SciencesNatural History MuseumCromwell RoadLondonSW7 5BDUK; ^3^ Department of Animal and Plant SciencesUniversity of SheffieldSheffieldS10 2TNUK; ^4^ Department of Biological SciencesMacquarie UniversitySydneyNSW2109Australia

**Keywords:** assumption, bias, caveat, Ornstein–Uhlenbeck, PCM, phylogenetic independent contrasts, trait‐dependent diversification

## Abstract

1. Phylogenetic comparative methods are becoming increasingly popular for investigating evolutionary patterns and processes. However, these methods are not infallible – they suffer from biases and make assumptions like all other statistical methods.
2. Unfortunately, although these limitations are generally well known in the phylogenetic comparative methods community, they are often inadequately assessed in empirical studies leading to misinterpreted results and poor model fits. Here, we explore reasons for the communication gap dividing those developing new methods and those using them.
3. We suggest that some important pieces of information are missing from the literature and that others are difficult to extract from long, technical papers. We also highlight problems with users jumping straight into software implementations of methods (e.g. in r) that may lack documentation on biases and assumptions that are mentioned in the original papers.
4. To help solve these problems, we make a number of suggestions including providing blog posts or videos to explain new methods in less technical terms, encouraging reproducibility and code sharing, making wiki‐style pages summarising the literature on popular methods, more careful consideration and testing of whether a method is appropriate for a given question/data set, increased collaboration, and a shift from publishing purely novel methods to publishing improvements to existing methods and ways of detecting biases or testing model fit. Many of these points are applicable across methods in ecology and evolution, not just phylogenetic comparative methods.

## Introduction

**Figure 1:**
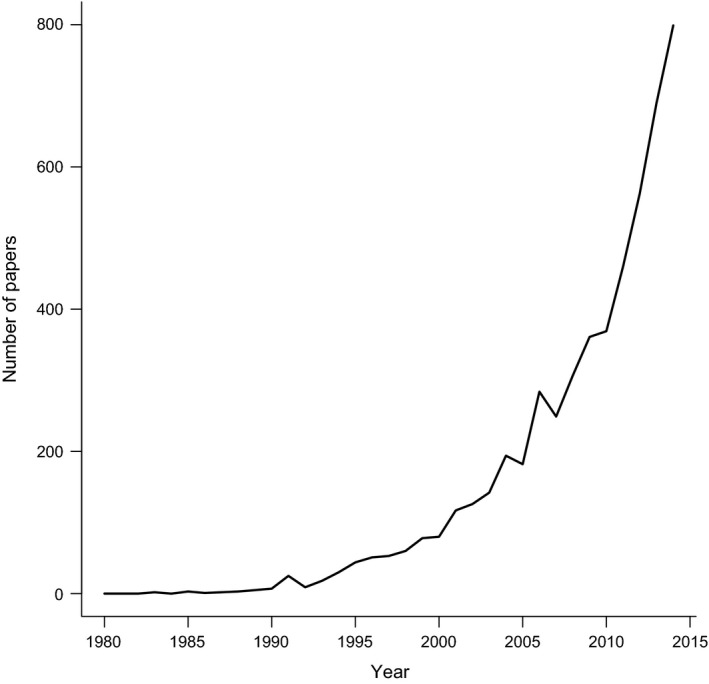
The number of papers containing the phrase ‘phylogenetic comparative’ published each year from 1980 to 2014 (Google Scholar search 13 April 2015). Data are available from figshare (Cooper, Thomas & FitzJohn 2016a).

Phylogenetic comparative methods (PCMs) were initially developed in the 1980s to deal with the statistical non‐independence of species in comparative analyses (e.g. Felsenstein 1985; Grafen 1989). Since then, PCMs have been extended to investigate evolutionary pattern and process (see reviews in O'Meara 2012; Pennell & Harmon 2013), and include methods for investigating drivers of diversification (e.g. Maddison, Midford & Otto 2007), the tempo and mode of trait evolution (e.g. O'Meara 2012), and models of speciation and extinction (e.g. Nee *et al*. 1994a). PCMs have also become extremely popular over recent years; the number of papers containing the phrase ‘phylogenetic comparative’ has increased dramatically since the 1980s (Fig. 1). With new methods being published almost weekly, there has never been a better time to be a comparative biologist.

Unfortunately, PCMs also have a ‘dark side’; they make various assumptions and suffer from biases in exactly the same way as any other statistical method – a fact that is well‐established in the literature (e.g. Freckleton 2009; Losos 2011; Blomberg *et al*. 2012; Boettiger, Coop & Ralph 2012). Increasingly, however, assumptions and biases are inadequately assessed in empirical studies, leading to poor model fits and misinterpreted results (see examples below). Additionally, little consideration is given to whether using a PCM is really appropriate for the question at hand (Westoby, Leishman & Lord 1995; Losos 2011).

We suggest that one cause of this problem is that although researchers developing and implementing new methods are aware of the limitations of their methods and the assumptions that underly them, this information is not always being effectively transferred to end‐users. Additionally, the tools and approaches used to fit models are often far more user‐friendly and better documented than the methods used to assess whether that model fit is reasonable. Clearly, more effort is needed to bridge the widening gap between those developing methods and end‐users. Here, we explore the causes of this communication gap and suggest some potential solutions. Note that many of these issues are applicable across methods in ecology and evolution, not just PCMs.

## Examples of the problem

Before exploring the reasons behind the communication gap, we give three brief examples of commonly used PCMs that have assumptions, biases or caveats that are often inadequately assessed in empirical studies. Because the aim of this paper was to provide positive ways to move forward, rather than to admonish authors for past errors, we do not cite papers we feel have fallen into these traps (especially as we are guilty of making some of the same mistakes).

### Phylogenetic independent contrasts

The phylogenetic independent contrasts method uses phylogenetic information to account for the fact that species in a comparative analysis are related to each other and thus may share similarities because they inherit them from their ancestors, not because of independent evolution (Felsenstein 1985; Harvey & Pagel 1991). This is the most commonly used phylogenetic comparative method (Felsenstein (1985) has been cited over 6000 times; Google Scholar search 9 December 2015), and a great deal of literature exists on the assumptions underlying the method, and ways of testing whether these assumptions are met. The method has three major assumptions (Diaz‐Uriarte & Garland 1996; although there are many more assumptions than these three) (1) that the topology of the phylogeny is accurate; (2) that the branch lengths of the phylogeny are correct; and (3) that traits evolve in the manner of the Brownian motion model, a simple model of trait evolution where trait variance accrues as a linear function of time (Cavalli‐Sforza & Edwards 1967; Felsenstein 1973). The third assumption is stated in Felsenstein (1985), although the other two are not explicitly mentioned. However, each assumption is explored in many subsequent papers (e.g. Felsenstein 1988; Grafen 1989; Harvey & Pagel 1991; Garland, Harvey & Ives 1992; Purvis & Rambaut 1995; Diaz‐Uriarte & Garland 1996; Hansen & Martins 1996; Martins & Hansen 1997; Freckleton 2000; Garland & Ives 2000; Hansen & Orzack 2005; Freckleton & Harvey 2006; Rohlf 2006). There are several ways of testing these assumptions, including looking for relationships among standardised contrasts and node heights (Grafen 1989; Freckleton & Harvey 2006), absolute values of standardised contrasts and their standard deviations (Garland, Harvey & Ives 1992; Diaz‐Uriarte & Garland 1996) and heteroscedasticity in model residuals (Purvis & Harvey 1995). These tests are fairly easy to implement and are included as standard model diagnostic plots in CAIC and the caper r package (Purvis & Rambaut 1995; Orme *et al*. 2013; R Core Team, 2015). However, the majority of studies using phylogenetic independent contrasts do not mention testing these assumptions (Freckleton & Harvey 2006; although they may have tested the assumptions and not recorded this). In addition, because phylogenetic independent contrasts are identical to phylogenetic generalised least squares models (Garland & Ives 2000; Rohlf 2006; Blomberg *et al*. 2012), these models also have the same assumptions that are equally rarely addressed.

### Ornstein–Uhlenbeck (single stationary peak) models of trait evolution

Most models of trait evolution are based on the Brownian motion model (Cavalli‐Sforza & Edwards 1967; Felsenstein 1973). The Ornstein–Uhlenbeck (OU) model can be thought of as a modification of the Brownian model with an additional parameter that measures the strength of return towards a theoretical optimum shared across a clade or subset of species (Hansen 1997; Butler & King 2004). OU models have become increasingly popular as they tend to fit the data better than Brownian motion models, and have attractive biological interpretations (Cooper *et al*. 2016b). For example, fit to an OU model has been seen as evidence of evolutionary constraints, stabilising selection, niche conservatism and selective regimes (Wiens *et al*. 2010; Beaulieu *et al*. 2012; Christin *et al*. 2013; Mahler *et al*. 2013). However, the OU model has several well‐known caveats (see Ives & Garland 2010; Boettiger, Coop & Ralph 2012; Hansen & Bartoszek 2012; Ho & Ané 2013, 2014). For example, it is frequently incorrectly favoured over simpler models when using likelihood ratio tests, particularly for small data sets that are commonly used in these analyses (the median number of taxa used for OU studies is 58; Cooper *et al*. 2016b). Additionally, very small amounts of error in data sets can result in an OU model being favoured over Brownian motion simply because OU can accommodate more variance towards the tips of the phylogeny, rather than due to any interesting biological process (Boettiger, Coop & Ralph 2012; Pennell *et al*. 2015). Finally, the literature describing the OU model is clear that a simple explanation of clade‐wide stabilising selection is unlikely to account for data fitting an OU model (e.g. Hansen 1997; Hansen & Orzack 2005), but users of the model often state that this is the case. Unfortunately, these limitations are rarely taken into account in empirical studies.

### Trait‐dependent diversification

Analyses of trait‐dependent diversification are used to detect whether particular traits promote high rates of diversification, leading to some clades becoming more diverse than others (Nee, May & Harvey 1994b). These kinds of analyses have been applied extensively in recent years to a variety of taxa and traits (e.g. Goldberg *et al*. 2010; Price *et al*. 2012; Givnish *et al*. 2014; Pyron & Burbrink 2014). Most use the binary state speciation and extinction model (BiSSE) and related methods (the original BiSSE paper Maddison, Midford & Otto (2007) has been cited 394 times; Google Scholar search 9 December 2015). However, Rabosky & Goldberg (2015) recently re‐evaluated the method and its assumptions and showed via simulations that a strong correlation between a trait and diversification rate can be inferred from a single diversification rate shift within a tree, even if the shift is unrelated to the trait of interest. They suggest that many examples of trait‐dependent diversification actually reflect this rate heterogeneity in trees and thus are biologically meaningless. Interestingly, these caveats are mentioned in earlier papers (Maddison, Midford & Otto 2007; FitzJohn 2010, 2012) but were seemingly not widely understood given the shock reaction to Rabosky & Goldberg (2015).

## What impedes information transfer about the limitations of PCMs?

These three brief examples illustrate that although the PCM community is aware of the limitations of PCMs, this information is not filtering through to everyone using the methods. Why might this be the case?

### Not everything is mentioned in the literature

As scientists, we mainly communicate our ideas through the literature. Unfortunately, some of the information needed to properly apply PCMs is not found in the literature. We refer to this knowledge as ‘PCM folklore’ because it tends to be passed down from PIs to graduate students, and within laboratories developing methods (and it is occasionally closer to fiction than fact!). Sometimes the folklore is based on best practice and includes tricks to get methods working, or useful rules of thumb; other times it is more opinion based, but over time these opinions become rules. Useful PCM folklore is often shared among developers, and among collaborating groups, but is not always shared outside of these circles. When it is shared, it tends to be as email exchanges of ‘dark advice’ that is not accessible to the rest of the community.

One example of PCM folklore is that species with studentised residuals ±3 are often omitted from regressions of phylogenetic independent contrasts, to avoid highly influential outliers affecting the results (e.g. Cooper *et al*. 2008). The rationale for this comes from Jones & Purvis (1997); however, the ±3 cut‐off is arbitrary, and barely mentioned in the original paper, but has become a rule of thumb for running these analyses (the paper has been cited >100 times, mostly as a justification of this procedure). Another example is in the defaults of programmes that perform PCMs. These often start out as arbitrary starting points for data exploration with no justification for their use, but over time become the way the analysis is always performed.

Other information about the limitations of a method may be absent from the literature due to the time‐lag between a new method being published and others having time to test it. For example, Felsenstein published the phylogenetic independent contrasts method in 1985 (Felsenstein 1985), but it was not until the early 1990s that thorough critiques of the method and its assumptions began to be published (e.g. Garland, Harvey & Ives 1992). This time‐lag is shorter with more recent methods for a number of reasons. First, historically theory and software papers were generally separated, whereas currently they are combined in the same papers making it easier to run simulations. Secondly, simulations testing methods are now required by journals (although simulations usually only show that the method behaves appropriately under ideal conditions), and finally, there are now far more people in the field and thus more papers published annually. However, even with this reduced time‐lag, we suspect there are still many hidden assumptions and biases in all PCMs, even established methods, that have yet to be properly explored in the literature. For example, see Maddison & FitzJohn's (2015) recent critique of Pagel's (1994) correlated evolution method, and Rabosky & Goldberg's (2015) discussion of trait‐dependent speciation models (Nee, May & Harvey 1994b; Maddison, Midford & Otto 2007).

### The literature is too technical and/or important details are difficult to locate

Although some information is not found in the literature (see above), the majority of assumptions and biases of PCMs are documented somewhere. A big issue for novice methods users (and often for advanced users too) is that this information can be extremely technical and dense. It is not unusual for papers to be long and full of equations. Of course, such detail is critical for describing methods and facilitates testing/assessment, implementation and future developments of the method. Additionally, in most cases equations could not simply be replaced with text. There is evidence, however, that heavy use of equations impedes understanding and communication of concepts in biology (Fawcett & Higginson 2012).

Another issue is that end‐users need to understand the assumptions and caveats of methods. Within many comparative methods papers, assumptions and caveats can be found in the Introduction, Methods, Results and/or Discussion of a paper – they are rarely neatly corralled in one place. The sheer volume of literature can thus become a barrier to understanding, even, and perhaps especially, for the best known methods. For example, we recently reviewed papers discussing the assumptions and limitations of phylogenetic independent contrasts (Felsenstein 1985), including how they are related to phylogenetic generalised least squares models (Garland & Ives 2000; Rohlf 2006; Blomberg *et al*. 2012). Even with prior knowledge of the key papers and authors to focus on, this resulted in ≈300 manuscript pages and a book to read to fully understand the method and its caveats. The volume of reading itself is perhaps not the key issue, rather it is assessing when you have reached sufficient understanding which may not be clear to all users.

The combined effects of a vast literature and sometimes opaque assumptions make it easy to miss pertinent details in PCM papers. These problems relate to due diligence for both end‐users and developers. Methods developers are not responsible for making sure that end‐users read the literature. Instead, the onus is on the end‐user to ensure that they have a clear understanding of the methods and caveats prior to using them. However, simple steps could be taken by methods developers, such as subheadings that point to caveats and assumptions, to add clarity and limit method misuse and misinterpretation.

### Users jump straight to the implementation of the method

In the early days of PCMs, some researchers provided stand‐alone packages to run their methods (e.g. PDAP; Diaz‐Uriarte & Garland 1996), others provided code in whichever language they chose to programme in (e.g. matlab code; Rohlf 2001), and still others provided no way of implementing their methods at all. This resulted in many frustrating hours (and days and months) trying to implement any new method you wanted to use. Writing your own implementation may be the best way to learn the intricacies of a method but is a major hurdle and can dissuade many potential users.

**Figure 2:**
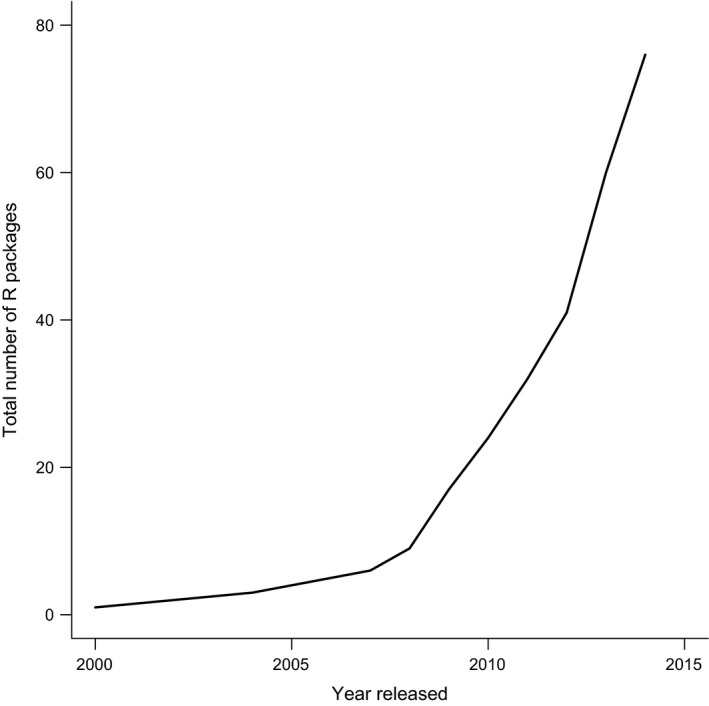
The cumulative total number of r packages for phylogenetics and phylogenetic comparative methods through time from 1980 to 2014. Source: Brian O'Meara's ‘cran task view: Phylogenetics, Especially Comparative Methods’ version 21 January 2015. Data are available from figshare (Cooper, Thomas & FitzJohn 2016a).

More recently however, the community has movedtowards mostly implementing methods in r (R Core Team, 2015;for a list of packages, see Brian O'Meara's ‘cran task view: Phylogenetics, Especially Comparative Methods’ https://cran.r-project.org/web/views/Phylogenetics.html), and code sharing has become almost ubiquitous. The number of r packages for PCMs has increased markedly since 2005 when APE (Paradis, Claude & Strimmer 2004) was released, and has increased particularly sharply since 2008 (Fig. 2). Simultaneously, more people are able to use r thanks to workshops and changes in student training; thus, when a new method is published, it is now possible to take an r package ‘off the shelf’ and use it to run the method immediately. The benefits of r are clear. It is available to all and has a wide and engaged support community, and perhaps most importantly, the source code for new methods is accessible. Users can fully explore any new method by examining the source code and running their own simulations. Indeed, Freckleton (2009) suggested that the ability to conduct PCMs in flexible computing environments such as r would improve our ability to implement methods correctly. However, the increasing use of r has instead led to more people (including the authors of this paper) jumping straight to the implementation of a method, without fully understanding what the method is doing, what its assumptions might be or what the results mean in a biological context. This is not the fault of methods developers and users should conduct due diligence in understanding a method before using it. Unfortunately, the problem is exacerbated by the fact that manuals, vignettes and help files for r packages rarely mention the assumptions of the method, or how to test model fit. The APE book (an excellent resource; Paradis 2011), for example, provides no guidance on assumption testing in its chapter on phylogenetic independent contrasts, even though the methods needed to do this are well‐established and very easily implemented in r (for a counter example, see CAIC and caper documentation; Purvis & Rambaut 1995; Orme *et al*. 2013).

## How can we solve some of these problems?

### Simplify, summarise and share

Many of the problems above arise because PCMs are difficult to communicate in purely descriptive terms. This is not entirely the fault of those writing these papers; most journals have strict word limits and a one line equation is generally a more succinct and precise descriptor of a method than a paragraph of text. As we note above, the onus is on the end‐user to read extensively and to do due diligence. Nonetheless, due diligence is a responsibility at all levels of research and that includes ensuring clarity to all target users, especially in describing assumptions and limitations.

One solution is to prepare an accompanying blogpost or video for each new method, explaining it inless technical terms. Some journals already encouragethis (including the British Ecological Society journals; see https://soundcloud.com/besjournals, https://www.youtube.com/user/MethodsEcolEvol), and even if they do not, there are great benefits to doing this anyway and hosting it on a personal website. The ability to share ideas with a non‐technical audience is a key skill to develop and may encourage more people to use the method. Some package developers also provide blog updates (e.g. phytools: http://blog.phytools.org; Revell 2012) that serve this function.

Encouraging increased efforts at reproducibility would also help make methods more accessible to new users, by providing fully worked examples that can act as a starting point for their analyses. Reproducibility can be achieved by including knitR (Xie 2015) reports in Supplementary Material showing exactly how each analysis was run (e.g. FitzJohn *et al*. 2014), or by requiring all analyses and code to be available on GitHub or Bitbucket. At the very least, having a list of the main assumptions, biases and caveats of the method somewhere obvious would reduce misuse and provide a place to point people to when they begin using a method (this will be difficult as there are often hidden assumptions in a method, and listing all possible assumptions and caveats may be unfeasible in some cases).

An important point here is that we do not see these possible solutions as entirely the responsibility of the method developer or package writer. The PCM community can help tooand indeed often does via platforms such as r‐sig‐phylo (http://www.mail-archive.com/r-sig-phylo@r-project.org/). However, the ability to find helpful replies on a listserv depends on the subject headings and details in the text. A longer lasting solution would be to make published methods papers, especially those that have a software focus, more readily updatable, rather than requiring a new paper for each update. One example that gets around this problem is PLoS Currents(http://currents.plos.org) where published papers can be updated relatively quickly with the full history of updated articles versioned.

Summarising the glut of literature that already exists for established methods is a more difficult problem to solve. The Oxford Bibliographies Evolutionary biology pages have lists of key papers (http://www.oxfordbibliographies.com/obo/page/evolutionary-biology), but these are curated by just a few individuals and tend to contain a lot of papers. One solution would be to establish a wiki‐style website where people could post summaries of commonly used methods, along with lists of key papers to read. The community would be responsible for peer reviewing these summaries to ensure all opinions are covered. Two excellent examples of a similar approach are Erick Matsen's Phylobabble discourse page for phylogenetics (http://phylobabble.org/), and the Prometheus wiki for protocols in plant physiology (http://prometheuswiki.publish.csiro.au/tiki-custom\_home.php). The British Ecological Society's Quantitative Ecology Special Interest Group is also in the process of creating a ‘Field Guide for Ecologists’ (http://bes-qsig.github.io/fge/) that will fill a similar niche in ecological methods. We plan to establish a similar guide for PCMs in the near future with help from across the community.

### Factors to consider before using a method

A key skill to develop in science is cynicism, that is never take results from PCMs (or any other statistical analysis) at face value. As the Manic Street Preachers (1996) put it, ‘Cynicism is the only thing that keeps me sane’. At a minimum, users should read the original papers describing a method, plus any recent updates, and look carefully for assumptions and caveats that may affect the analyses at hand. A good way to check a method is to simulate some data and see whether the results are as expected (e.g. Boettiger, Coop & Ralph 2012). This can expose hidden assumptions or biases that have not been explored in the papers accompanying the method, or reveal a lack of understanding of the mechanics of the method being used. It is also important to determine whether the method will work on a particular data set. One key consideration is how many species are required for reasonable power. Often methods require more species than are usually available. For example, the new trait‐dependent diversification method of Rabosky & Huang (2016) is ‘primarily applicable to phylogenies that include at least several thousand tips’ although the authors suggest that most empirical analyses have <1000 tips. Other considerations include whether the method is influenced by polytomies and whether the method is applicable to both ultrametric and non‐ultrametric trees. Indeed, many methods arbitrarily resolve polytomies using zero length branches; thus, polytomies can inflate rates of evolution, and bias models of evolution (Cooper & Purvis 2010). Some current implementations of the OU model should not be used with non‐ultrametric trees (e.g. MOTMOT; Thomas & Freckleton 2011) because they are based on transforming the tree directly, rather than transforming the variance covariance matrix. The problem is that where there is a pair of tips and at least one tip does not survive to the present, the expected covariances relating each of those two tips with any other tip in the tree are not identical. Worked examples and explanations are provided in Slater (2014). Although this is not a problem with applying the OU model to non‐ultrametric trees *per se*, it is an example of different implementations of a common model that some users may not be aware of.

It is also important to avoid retrofitting questions to the newest methods; instead, we should think carefully about the question, whether the method is appropriate for the question, and whether PCMs are needed at all (Westoby, Leishman & Lord 1995; Losos 2011). In some cases, editors and reviewers may suggest using PCMs where they are not appropriate, and users should feel confident in rejecting these suggestions. Finally, end‐users should never be afraid to question standard practice, sometimes it is just PCM folklore.

### Solutions and incentives

It is important to recognise that our ability to do rigorous quantitative science often relies on highly skilled methods developers, especially as evolutionary biology becomes ever more computationally intensive. We cannot afford to lose these people to industry, nor can we afford to pay industry wages; thus, we need to make it worthwhile for such skilled researchers to remain in (or at least interact with) academia.

First we need to stop insisting that methods papers are entirely novel. Improvements to existing methods, and ways of detecting biases or testing model fit should be sufficient for publication. This is fairly standard in other fields, for example statistical phylogenetics, and these kinds of papers are arguably more useful to the community than constantly publishing new methods. When novel methods are published, journals should encourage researchers to include lists and ways of testing the assumptions of their methods within the original publications and packages, and request simplified summaries to accompany technical papers.

Secondly, we need to fund pure methods development, including incremental methods. Currently, it is difficult to get funding for purely methods driven research; an empirical component is generally needed and methods development is often seen as part of the bigger empirical picture, rather than the reverse (although it is also hard to get funding for empirical projects).

Finally, an obvious solution to many of these problems is for methods developers and end‐users to collaborate more. Both parties can benefit greatly by collaboration. Some benefits are obvious; for example, methods developers can gain extra data sets to test their ideas on and people who will discover corner cases and bugs in their software before it gets released, whereas end‐users can work with the most cutting edge methods and software. Most methods are not designed in a vacuum; they have a specific purpose usually based on predictions from theory, experimentation or observation. There is a huge benefit in sharing ideas as well as products (data and code) and time, as long as the benefits to both parties are not heavily asymmetric.

## Conclusion

We are currently in an exciting period for phylogenetic comparative methods research. New methods are being published with increasing regularity, and we are also beginning to question older methods and classical ways of looking at comparative questions. In addition, the field is becoming more open, with code being shared before analyses are even submitted for publication, and collaborative software development across groups, and even continents, is becoming more common. However, while embracing these changes, we also need to ensure that we do not forget that PCMs have assumptions, caveats and biases like every other method. These need to be highlighted so they can be accounted for in empirical analyses, and we need to be more active at providing ways of assessing these issues when publishing new methods. As members of the phylogenetic comparative methods community, we have a responsibility to find innovative ways to tackle these challenges.

## Data accessibility

All data from Figures 1 and 2 are available on figshare: https://dx.doi.org/10.6084/m9.figshare.2057802.v1. r code for recreating the figures is available on GitHub: https://github.com/nhcooper123/pcm-darkside.
